# Sensory Acceptance and Physicochemical Properties of Beef Meatballs Fortified With Apple ( *Malus domestica* ) Pomace

**DOI:** 10.1002/fsn3.70955

**Published:** 2025-09-12

**Authors:** Peter Gracey, Olga I. Padilla‐Zakour, Elad Tako

**Affiliations:** ^1^ Department of Food Science Cornell University Ithaca New York USA

**Keywords:** apple pomace, fiber, polyphenols, processed meat, sensory analysis

## Abstract

Introduction: Apple pomace (AP), a byproduct of apple processing, generates over 4 million tons of global waste annually. Its high moisture content and organic load pose environmental concerns, while disposal imposes financial costs. However, AP is rich in dietary fiber, antioxidants, and micronutrients, offering potential as a functional ingredient to enhance the nutritional quality of food products. This study evaluated the feasibility of incorporating freeze‐dried AP into beef meatballs at two levels (10% and 20% *w/w*) and assessed its effects on sensory attributes, texture, color, and cooking performance. Methods: Pomace from three apple varieties—Cortland, Empire, and Red Delicious—was freeze‐dried for 24 h and analyzed for polyphenol and fiber content. The rehydrated pomace was then added to 80% lean beef at 10% and 20% inclusion rates. Meatballs were evaluated for texture, color, and proximate composition. A sensory panel of 104 untrained consumers assessed aroma, texture, taste, and overall preference. Data were analyzed using Friedman's two‐way analysis. Results: No significant differences (*p* > 0.05) were found in sensory attributes or consumer preference among treatments. Texture analyses also showed no significant variation (*p* > 0.05). There were significant differences (*p* < 0.05) between the internal colors of the meatballs. The 20% inclusion treatment had the lowest cooking yield both post‐cooking and after 1 day of refrigerated storage. Significance: Results support the feasibility of incorporating up to 20% apple pomace into meat products without compromising sensory acceptability. AP offers a high‐fiber, sustainable ingredient option for value‐added meat applications. Future studies should explore broader applications and potential health benefits.

## Graphical abstract

Using apple pomace to fortify beef meatballs .

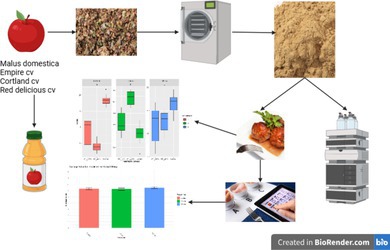

## 1 Introduction

In 2023, 97.34 million metric tons of apples were produced globally; the US produces 6.2% of the world's apples (USDA 2023; Villa 2025). While most consumers prefer fresh apples, about 35% of apples undergo processing. Apple juice production is the primary use for processed apples, though they are also made into jams, jellies, cider, vinegar, and dried products. With a juice extraction efficiency of roughly 75%, the industry generates 25%–30% of the fruit as waste (Jackson et al. 2022). Apple pomace (AP) is a waste product of apple juice and cider production. It constitutes the leftovers of this process, including seeds, skin, flesh, stem, and core, currently used for fertilizer, animal feed, or landfill application (Lyu et al. 2020). These are not sustainable solutions for this waste product, as AP has a high moisture and biodegradable organic load that allows for fermentation to take place rapidly (Bhushan et al. 2008). This rapid fermentation caused by anaerobic bacteria produces methane, which is a greenhouse gas that contributes to climate change and ozone depletion (Mohamed Raimi et al. 2020). The nutrient composition and moisture content of AP can vary based on the variety of apples used and the kind of processing the apples went through (Karwacka et al. 2023).

The moisture and nutritional content is what causes the rapid fermentation at landfill and reduces the ability of the AP to be transported, while maintaining the nutritional value (Antonic et al. 2020). Freeze‐drying is a common process for extending the shelf life of fruits, as it has been shown to reduce the rate of enzymatic browning, while maintaining the structure of bioactive compounds (Donno et al. 2019; Rawson et al. 2011). Juice and cider producers use a blend of apples that produce the desired taste characteristics (Valois et al. 2006). In the current study, a blend of Empire, Cortland, and Red Delicious apples was selected for pomace production to reflect typical commercial practices. These cultivars are widely grown, commonly used in juice and sauce production, and offer a representative composition of sugars, acids, and polyphenols (Lyu et al. 2020). Using a blend enhances the relevance of the study to food manufacturers who would be sourcing pomace from mixed‐apple processing streams.

The need to upcycle organic waste products has had many approaches, and one of the options is to incorporate products like AP into food products, for example, processed meat‐based products. Meatballs are a practical and industry‐relevant meat‐based product to test functional ingredients such as pomace. The USDA has outlined in the labeling policy for meat‐based products that meatballs require a minimum of 65% meat, allowing up to 35% of the formulation to include nonmeat ingredients (Agriculture, 2024). This allows for flexibility in the inclusion of functional ingredients like AP.

Beyond waste reduction, there are broader health and environmental motivations for reformulating meat products. Excess processed red meat consumption has been linked with the incidence of certain cancers like colorectal and stomach, as well as type 2 diabetes and heart disease (Farvid et al. 2021; Humans 2018; Research 2018). Gerber et al. (2013) reported that, from livestock supply chains, 14.5% of global greenhouse gas emissions originate, with a significant contribution to methane production. A potential approach is to reduce the reliance on animal‐derived ingredients in meat products by incorporating plant‐based components (Pintado and Delgado‐Pando 2020).

Springmann et al. (2018) estimated that reducing processed meat consumption to zero and limiting red meat to one serving per week, while emphasizing plant‐based foods in high‐income countries, could lower greenhouse gas emissions by 60%. Increasing plant‐based ingredients in food could enhance dietary fiber intake while reducing the meat content, impacting two main issues of feeding the future population: global malnutrition and protein availability (González et al. 2020). This is an alternative that may allow consideration by consumers looking to reduce their meat consumption, as long as the sensory characteristics remain acceptable (Lang 2020).

To ensure such alternatives are both appealing and effective, functional ingredients can be incorporated to enhance product stability and quality (Broucke et al. 2025). A functional ingredient is an ingredient that is intentionally added to a food for a specific function, for example, antioxidants to reduce lipid oxidation or antimicrobials if a food product requires microbes to be inhibited or killed (Galanakis 2021; Garti and McClements 2012). AP is regarded as a functional ingredient due to the health properties associated with the fiber content (pectin), polyphenols such as flavanols, and micronutrients present within the by‐product (Kammerer et al. 2014; Skinner et al. 2018). AP has already been widely studied as a potential functional ingredient in beef burgers (Bastos et al. 2014; Pollini et al. 2022; Shin et al. 2017), ground pork, chicken thighs (Khodaei et al. 2023), salami (Grispoldi et al. 2022), buffalo sausages and patties (Younis and Ahmad 2015; Younis and Ahmad 2018). However, there is limited research on the incorporation of AP into commercially relevant processed meat products, particularly beef meatballs, despite their suitability for functional ingredient inclusion and widespread consumer acceptance. To address this gap, the present study investigated the composition of apple pomace and evaluated its impact at varying inclusion levels on the physicochemical properties, cook yield, and sensory acceptance of beef meatballs. This approach aims to assess the potential of AP as a sustainable and functional ingredient in processed meat formulations.

## 2 Materials and Methods

### 2.1 Preparation of Apple Pomace

All three varieties of apples (Cortland, Red Delicious, and Empire) were bought in bulk from P&C Fresh in Ithaca, NY. They were then transported on the same day to the Cornell Food Venture Center in Geneva, NY, and then processed using a Goodnature commercial juice press (X‐1 Mini, Buffalo, NY). The resulting AP after juice extraction was then placed on trays and freeze‐dried for 48 h, with 9 stages of vacuum and temperature treatment. The settings used can be seen in Figure S1 (Millrock MX53 Freeze dryer, Kingston, NY). After freeze‐drying, the AP was then milled 30 g at a time using a coffee mill until a consistent particle size was reached (Jura‐Capresso Inc., Montvale, NJ). The pomace was then stored in freezer bags in a cool, dry place (17°C) outside of direct sunlight, until use.

### 2.2 Analysis of Apple Pomace (AP)

#### 2.2.1 Placeholder TextChemicals

All solvents, including ethanol, methanol, acetonitrile (chromatography quality), hydrochloric acid, and further chemicals and consumables, including for UV–vis analysis, were purchased from VWR International (Radnor, PA, USA). Deionized water was obtained by the Purelab Flex system (Elga Labwater, Woodridge, IL, USA). HPLC reference standards, gallic acid mono hydrate (CAS 149–91‐7, ≥ 97.5%), caffeic acid (CAS: 331–39‐5, ≥ 95%), (+)‐catechin (CAS: 225937–10‐0, ≥ 98%), (−)‐epicatechin (490–46‐0, ≥ 99%), quercetin hydrate (CAS: 522–12‐3, ≥ 90%), quercetin dihydrate (6151‐25‐3, ≥ 98%), were purchased from Sigma Aldrich (St. Louis, MO, USA), and malvidin‐3‐O‐glucoside chloride (7228‐78‐6, ≥ 95%) was purchased from Indofine Chemical Company (Hillsborough Township, NJ, USA).

#### 2.2.2 Placeholder TextApple Pomace (AP) Extraction

The AP was extracted for polyphenol composition analysis, as was previously described by Pollini et al. (2021). Briefly, a lyophilized sample of (AP) (100 g) was extracted with 1500 mL of 50% ethanol solution, which was adjusted to a pH of 2.0, with the container covered with aluminum foil to minimize light exposure. The mixtures were agitated for 12 h at room temperature (21°C ± 1°C) on a shaker and subsequently centrifuged at 4000 RPM for 10 min. The supernatants were then filtered through a 0.45 μm cellulose acetate filter and stored at 5°C until further analysis.

#### 2.2.3 Analysis of Phenolic Compounds by HPLC

Prior to HPLC analysis, extracts were passed through Captiva Filter Vials (0.2 μm, Agilent, Santa Clara, CA, USA). The analysis was conducted using an HPLC coupled with a Diode Array Detector (DAD). The employed method was adapted from Peng's method (Peng et al. 2002). The system was an Agilent 1200 Infinity Series (degasser G1379B, autosampler G1367C, column compartment 1316B, DAD G1315C with standard flow cell, Agilent Technologies, Santa Clara, CA, USA). A precolumn (Zorbax Eclipse XDB C18 4.6 × 12.5 mm, 5 μm) was coupled to an analytical column Zorbax Eclipse (XDB‐C18, 4.6 × 50 mm, 1.8 μm, both from Agilent Technologies Santa Clara, CA, USA), with two mobile phases of water/phosphoric acid (99.5:0.5 v/v) as Phase A and acetonitrile/phosphoric acid (99.5:0.5 v/v) as Phase B. The flow was set to 1.0 mL/min, and the temperature at 50°C. The binary gradient of the mobile phases was set as following proportions (percentage of Phase A): 0 min 95%, at 10 min 81%, 10.25–12.5 min 67%, 13–15 min 5%, and 15.5–19.5 min 95%. The injection volume was 4 μL for all samples. The calibration curve was based on 6 points covering the range of 1.0–200 mg/L for all compounds except catechin, with a range of 1.0–1000 mg/L. All compounds were quantified by their corresponding standards, except polymeric tannins, which were expressed in catechin equivalence, quercetin glycosides, which were expressed in quercetin equivalent, and total anthocyanins, which were expressed in malvidin 3‐O‐glucoside equivalent.

#### 2.2.4 Other Chemical Analyses

Malic, tartaric, and citric acids were quantified using an HPLC method adapted from Schneider et al. (1987). Total polyphenolic content was evaluated using the Folin–Ciocalteu assay (Lamuela‐Raventós 2018). The levels of glucose, fructose, and sucrose were determined through enzymatic assays as described by Mccloskey et al. (1984). The fiber analysis was completed according to AOAC method 991.43 with modifications for the AP substrate (Ferjančič et al. 2022).

### 2.3 Placeholder TextPreparation of Meatball Treatments

**FIGURE 1:**
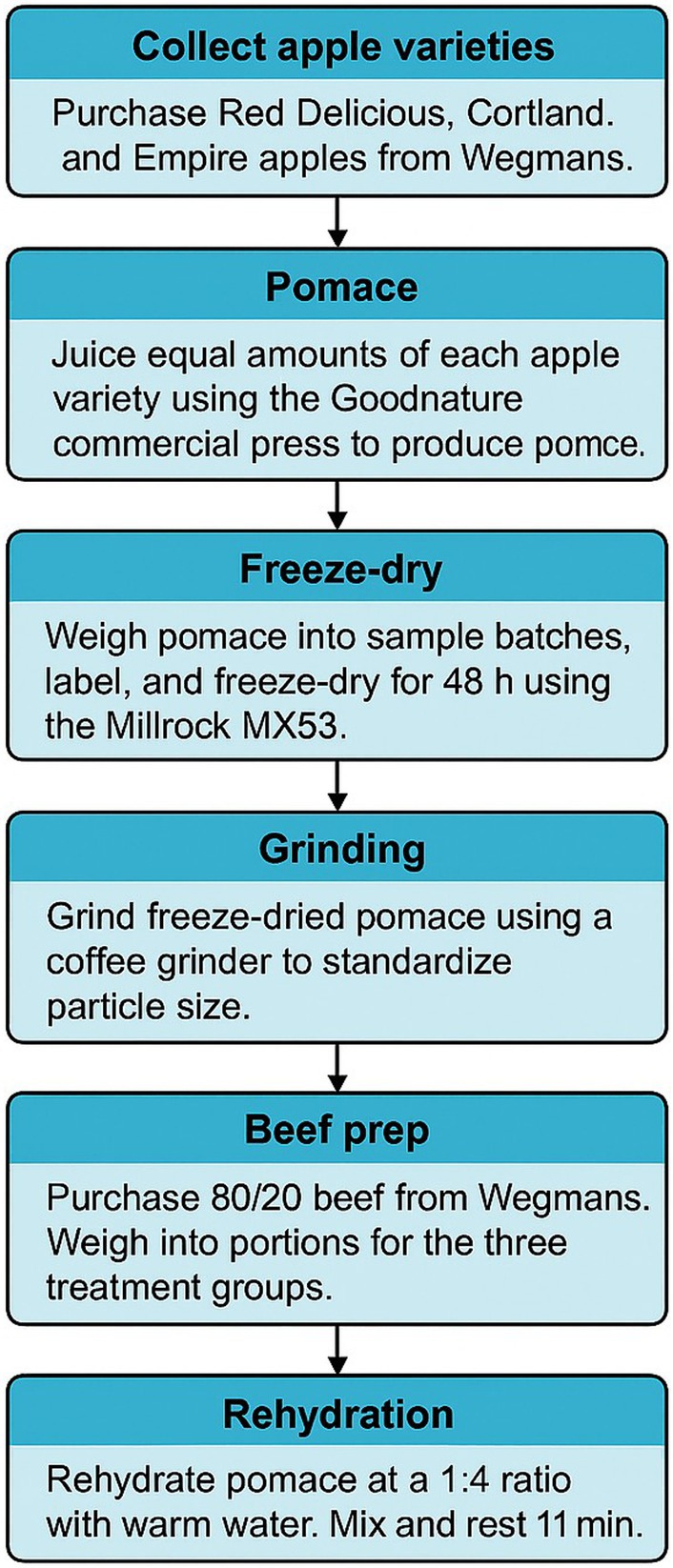
Workflow for the preparation of beef meatballs with apple pomace for sensory and instrumental analysis.

The ground meat was purchased the day before the preparation of the meatballs from Wegmans supermarket in Ithaca, NY. The ground beef was 80% lean and 20% fat. The meat was stored in a refrigerator at 4°C ± 1°C overnight. The ground beef was taken out of the refrigerator and weighed to the correct portions required for the three treatment groups, with the weights and percentages outlined in Table 1. The % AP utilized was based on preliminary trials (10%–40% addition) that identified 10% and 20% as preferred options. The AP was hydrated in separate batches as needed, using 40°C water at a ratio of 1:4 AP to water. The mixture was stirred thoroughly with a spoon in a bowl to ensure even hydration and allowed to rest for 10 min. Sodium chloride (2% *w/w*) was then added while the food processor (KitchenAid mixer, Greenville, OH, USA) mixed the beef and hydrated AP. After 1 min of mixing, the spatula was used to scrape the meat off the flat beater used in the mixer. The meat was then mixed for a further min, the bowl was removed, and the mixture was weighed out at 20 ± 1 g portions that were hand rolled and placed onto trays lined with baking sheets. The three different formulations were placed on separate baking sheets and stored in the fridge (4°C ± 1°C) overnight (12 h). The flowchart of the processing of the AP and meatballs can be seen in Figure 1. This same process was used to make every batch for sensory analysis, proximate analysis, and texture profile analysis.

**TABLE 1:** Formulations used in the preparation of meatballs with apple pomace (AP).

Ingredient (g)	Control	10% w/w (AP)	20% w/w (AP)
Ground beef	2178	2225	2607
Salt	22	25	33
Apple pomace dry + water = weight added	N/A	50 + 200 = 250	132 + 528 = 660
Total weight of meatball batch	2200	2500	3300

*Note:* This was the formulation of the batch used for sensory evaluation.

### 2.4 Sensory Analysis for Consumer Acceptance

The meatballs were taken from a refrigerator after overnight storage at 4°C. The meatballs, once set up on the tray with even spacing, were allowed to rest at room temperature (21°C ± 1°C) for 10 min before they were put into the oven. The meatballs were oven‐roasted at 175°C for 20 min. Once cooked, the meatballs were checked with a thermometer (Taylor 1476, Oak Brook, IL, USA) to make sure they met the 71°C, which is the safe minimum internal temperature for ground meats outlined by the USDA (USDA 2020). They were then placed into trays with aluminum foil that had a water bath underneath and heat lamps above to maintain 60°C until they were ready for evaluation. The meatballs were served in white paper baskets labeled with a randomly assigned 3‐digit identification number for the treatments. To cleanse the palate between samples, the participants were asked to consume water. The participants were given a random order to evaluate the three treatments of meatballs in a single session. The questions that were asked are outlined in Figure S7.

#### 2.4.1 Participants and Ethical Considerations

A total of 104 participants were recruited from the Cornell University campus community via email and posters. The average age of participants was 33 years, with a gender distribution of 47% male and 53% female. Familiarity with the product was not required; however, participants were informed that the product contained beef to discourage vegetarians from participating. The sensory evaluation was conducted at the Sensory Analysis Centre at the Department of Food Science at Cornell University, Ithaca, NY. The study was approved by the Cornell University Institutional Review Board for Human Participants (protocol #1405004676, reviewed in 2024). The testing procedures and facilities complied with ISO 8589 standards for sensory analysis (ISO, 2007). Red Jade sensory software (Curion, Deerfield, IL, USA) was used for data collection. Participants were presented with a digital informed consent form at the beginning of the study; only those who signed the form were permitted to continue. All responses were anonymized and stored in password‐protected files to ensure data confidentiality.

### 2.5 Texture Profile Analysis

For the objective analysis, an identical procedure was followed to make a new batch of the three treatment groups of cooked meatballs. To objectively assess the texture of the meatballs and have numerical data on specific characteristics, a texture analyzer (TA. XT plus model, Stable microsystems, Godalming, Surrey, UK) was used to measure hardness, cohesiveness, and chewiness. Texture profile analysis was conducted using a 38 mm cylindrical probe with a 5 g trigger force. Samples were compressed to 15% of their original height in two consecutive cycles, with a test speed of 1.0 mm/s and a 5‐s interval between compressions. Measurements were completed in quintuplicate (*n* = 5) for each of the three treatment groups. The following parameters were recorded: (I) Hardness is the peak force that occurs during the first compression; (II) Cohesiveness is a value that can describe how well the product withstands a second deformation relative to its resistance under the first deformation; (III) The chewiness of solids is the hardness multiplied by the cohesiveness multiplied by the springiness of the product.

Hardness=maxforce of first compression

Cohesiveness=Area2/Area1

Chewiness=Hardness×Cohesiveness×Springiness

### 2.6 Cook Yield Analysis

This analysis was conducted on 10 meatballs from each treatment. The samples were weighed using an Ohaus scale (PR5201N/E, Parsippany, NJ, USA) at room temperature (21°C ± 1°C) before and immediately after cooking. The equation below was used to calculate the yield percentage of each sample (Gök et al. 2011; Grasso et al. 2019).

$$\text{Cooking Yield}\;\left(\%\right)=\left(\text{Cooked weight}/\mathrm{Raw}\;\text{Weight}\right)\times 100\%$$

### 2.7 Color Analysis

The Hunter Lab system was used to assess the color of 5 samples of cooked meatballs from each treatment, after allowing them to equilibrate to room temperature (21°C ± 1°C). The measurements were one color measurement from the outside; the meatball was then cut in half, and two measurements were taken, the center of the meatball on both sides, and all results were noted. The measurements were taken with a calibrated Konica Minolta Chroma meter CR‐400, and the tile used to calibrate was also from Konica Minolta with a consistent light source at a 2° angle (Konica Minolta Sensing Inc., Tokyo, Japan). The formula below was used to calculate the difference in color between the samples and between treatments Anger (1977).

$$∆E={\left[{\left(L^{*}-L_{0}^{*}\right)}^{2}+{\left(a^{*}-a_{0}^{*}\right)}^{2}+{\left(b^{*}-b_{0}^{*}\right)}^{2}\right]}^{0.5}$$

### 2.8 Proximate Analysis and pH of Meatball Treatments

The proximate analysis was completed on one 80 g cooked sample from each treatment group. For the analysis of dry matter and moisture, the protocol included a two‐step drying process to ensure that both measurements could be attained accurately. The first drying step used an oven at 60°C overnight, and then the oven‐dried samples were milled, and the residual moisture was calculated using AOAC 930.15 (Thiex 2019).

The pH procedure involved 15 g of the wet sample mixed with 200 mL of deionized water and allowed to stabilize for 5 min. The samples were then analyzed using a Thermo Orion Combination Sure Flow electrode with a pH meter (Ohaus, Aqua searcher, Montvale, NJ, USA).

Crude protein was measured by the AOAC method 992.15 (McClements et al. 2021). From there, the combustion instrument is standardized with EDTA, and subsequently the samples were tested, and the results of the combustion were used in the equation below.

Crude protein,%=nitrogen,%×6.25

The Ash content was measured according to AOAC 942.05 (Thiex et al. 2019). Briefly, 2 g of meat was placed into a crucible and moved into a furnace at 600°C. The sample was then held at this temperature for 2 h and then placed into a desiccator to cool, and the sample was weighed immediately. The percentage of ash was calculated using the equation below:

$$\begin{array}{c} \mathrm{Ash}\%\left(w/w\right)=\left(\text{weight of test portion}\;g-\text{weight loss}\;\mathrm{on}\;\text{ashing}\;g/\right.\\ \;\left.\text{weight of test portion}\;g\right)\times 100 \end{array}$$

Crude fat was measured using the ANKOM system (ANKOM Technology, Macedon, NY, USA), which uses a hydrochloric acid (HCL) procedure that includes a filter bag technique filled with sample and then hydrolyzed with HCL for 60 min in a sealed Teflon vessel. Final extraction was performed with solvent at 90°C for 60 min using the ANKOM XT15 Extractor (ANKOM Technology, Macedon, NY, USA). Total Fat content was determined by loss of weight.

### 2.9 Statistical Analysis

Proximate composition and pH measurements were conducted by Dairy one analytical services (Ithaca, NY, USA). Crude fiber, crude protein, and crude fat were analyzed in duplicate to confirm the accuracy of the third‐party results. Separate batches were prepared for three distinct purposes: (1) sensory evaluation with 104 participants, (2) third‐party compositional analysis (including crude fiber, crude protein, moisture, ash, pH, crude fat, and dry matter), and (3) objective analyses (color, texture, and yield).

Objective data (color, texture, and cook yield) were analyzed using general linear models in R. For external color and yield, one‐way ANOVA models were constructed, with treatment group as a fixed effect. For internal color, linear mixed‐effects models were used. In all cases, estimated marginal means were obtained, and Tukey‐adjusted pairwise comparisons were conducted. The null hypothesis of no treatment effect was rejected at *p* < 0.05.

Sensory data (appearance, aroma, flavor, mouthfeel, and overall liking) were collected on a 9‐point hedonic scale for each treatment (Control, 10%, 20% AP). As the data were ordinal and participants rated all treatments, a Friedman test was used to assess differences across treatments for each attribute. All statistical analyses were conducted in R using the rstatix package for the Friedman test. A significance level of *α* = 0.05 was used for all tests.

There was also statistical analysis done on the mean rank scores of the different treatment groups. Friedman's two‐way ANOVA, a nonparametric test, was conducted as the data were ordinal, repeated‐measure data.

## 3 Results and Discussion

### 3.1 Chemical Composition of Apple Pomace (AP)

Results of Table 2 include the analysis of the AP, which includes fiber, organic acids, sugar content, and polyphenol analysis. The dietary fiber was measured as 40% for the dehydrated AP. The titratable acidity of the AP was 0.26 mg/100 g, with the only detectable organic acid being malic acid, measured at 1242 mg/100 g using an enzymatic assay. Fructose was the most abundant sugar in the sample, measured at 2.85 mg/100 g using the HPLC method. In terms of polyphenol content, the most abundant was polymeric tannins at 401.5 mg/100 g, with flavanols being measured at 67.34 mg/100 g.

**TABLE 2:** Chemical composition of freeze‐dried apple pomace (AP).

Parameters measured	Measurement
*Fiber*	*(% w/w)*
Total Dietary Fiber^1^	40
*Organic acids and acidity*	*Weight based concentration (mg/100 g)*
Malic acid^2^	82.8 ± 0.5
Titratable acidity^3^	170 ± 10
*Sugar content*	*Weight based concentration (g/100 g)*
Sucrose^4^	0.24 ± 0.05
Glucose^4^	0.76 ± 0.1
Fructose^4^	1.90 ± 0.1
Total sugars^4^	2.89 ± 0.1
*Polyphenolic compounds*	*Weight based concentration (mg/100 g)*
Caffeic acid^4^	1.70 ± 5
Polymeric tannins^4^	401 ± 5
Protocatechuic acid^4^	20.0 ± 5
Syringic acid^4^	29.0 ± 5
Coumaric acid^4^	45.0 ± 5
Ethyl gallate^4^	34.0 ± 5
Ferulic acid^4^	35.0 ± 5
Total flavanols^5^	448.90 ± 5
Tannin (GAE) ^6^	2040 ± 250

*Note:* The superscript numbers beside the parameters measured indicate which method was used to ascertain these results. 1 indicates the AOAC 991.43 Mod., 2 refers to an enzymatic assay, 3 is titration, 4 is high‐pressure liquid chromatography, 5 quercitrin equivalence, 6 Folin–Ciocalteu Assay Gallic Acid eq. (GAE).

As previously demonstrated, the polyphenol content of AP can vary depending on the apple variety and seasonal weather conditions. However, a consistent finding across studies is that AP contains high levels of both polyphenols and dietary fiber (Antonic et al. 2020). This is further evidenced by the results shown in Table 2, as these values demonstrate that the AP produced in this study is comparable in fiber and polyphenol content to previous work (da Silva et al. 2020; Dietrich et al. 2004; Kalinowska et al. 2020; Xu et al. 2016).

Fiber has been seen to be lacking in Western diets, as it was found in a study done by Miketinas et al. (2023) that only 4% of US adults meet the adequate intake for dietary fiber. A 40% Dietary Fiber measurement as seen in Table 2 is important for the current study, as the nutrient that is lacking in meat products is fiber (Das et al. 2020). Incorporating an ingredient containing 40% dietary fiber adds nutritional value to both the ingredient itself and the meatball, enhancing its appeal to consumers. This 40% dietary fiber result is comparable to other upcycled materials, for example, the pea tuber that usually goes to landfill has 51% dietary fiber and varieties of orange bagasse can have a dietary fiber percentage of 63% (Huang et al. 2019; Mejri et al. 2019). The impact on intestinal and overall health from the increased consumption of dietary fiber can be beneficial, as diets higher in fiber have been shown to be beneficial for gut motility, body weight, and abdominal adiposity, as well as the benefits to insulin sensitivity (Barber et al. 2020; Ioniță‐Mîndrican et al. 2022).

The malic acid that is present in the AP has had multiple studies aimed to investigate how the inclusion of this organic acid could improve the safety of foodstuffs. Recent findings demonstrate that malic acid can significantly enhance the antimicrobial efficacy of pressure‐based decontamination treatments. For example, after a 48‐h habituation period, treatment at 300 MPa and 4.4°C alone resulted in a modest 0.45 log reduction of STEC after 5 min; however, when combined with malic acid, the reduction increased to 4.26 log cycles (*p* < 0.05), highlighting its potential as an effective antimicrobial adjunct (Ghimire et al. 2025). These treatments to reduce bacterial loads also do not affect the quality of the beef at the point of sale (López‐Fernández et al. 2022; Mani‐López et al. 2012). Therefore, this suggests that there could be safety benefits to the inclusion of AP in beef meat‐based food products, as well.

Table 2 also includes the polyphenols measured in the freeze‐dried AP, with the highest value obtained from polymeric tannins, flavanols, and ferulic acid. Fresh apples have been tested in other studies for their polyphenol contents, and there are some polyphenol groups that have remained consistent throughout testing; for example, flavanols, hydroxycinnamic acids, and anthocyanins (Feng et al. 2021). The variation between pomace polyphenol results in this study and the results from previous studies was expected to be different as the polyphenol abundance can differ between tested apple varieties, harvest time, and processing method (Guyot et al. 1998; Kschonsek et al. 2018; Tsao et al. 2003). Li et al. (2020) utilized a very similar method to what was conducted in this study for processing the AP. Li et al. (2020) focused on improving the extraction process, but they also found two common polyphenols—caffeic acid and ferulic acid—which is encouraging given polyphenol content variations between apple varieties. The mentioned polyphenols being a consistent staple in AP, and despite the freeze‐drying process, provides validity to the idea that AP is a beneficial food ingredient to consumers.

### 3.2 Physiochemical Composition of the Meatball Treatments

The research by Barthélémy et al. (2024) demonstrated that the reformulation of food could be an opportunity to improve the nutritional status at the population level. Table 3 outlines the analysis completed on the three treatment groups of the meatballs. The fiber in the meatballs increases due to the fiber present in the AP. As expected, the moisture and the acidity increased as the proportion (%) of hydrated AP that was included in the meatball increased. The moisture content percentage increased from 55% in the control to 58% when 20% of the meat was replaced with AP. The pH of the tested meatball samples decreased from 6.2 in the control treatment to 6 in the 20% AP treatment group. The results of the crude fat were within a percentage point, with the lowest measurement being from the 10% AP inclusion at 15.17%. The highest was from the 20% AP inclusion at 16.54%.

**TABLE 3:** Proximate composition of meatball with apple pomace (AP) addition.

Parameter measured	Control	10% AP	20% AP
Fiber (%)	1.80	1.90	2.20
Moisture (%)	55.00	54.60	58.00
Dry Matter (%)	45.00 ± 16.65	45.40 ± 16.8	42.00 ± 15.54
Crude Protein (%)	27.40 ± 7.12	24.00 ± 6.24	20.00 ± 5.2
Crude Fat (%)	15.85 ± 7.29	15.17 ± 6.98	16.54 ± 7.61
Ash (%)	2.52 ± 0.73	2.24 ± 0.65	2.09 ± 0.61
pH	6.20 ± 0.31	6.10 ± 0.31	6.00 ± 0.3

*Note:* Statistical analysis could not be conducted for Table 3 due to the data being provided by a third party in summarized form only (mean ± assay error), without access to raw replicate values. As a result, standard errors reflect analytical method accuracy rather than biological replication.

The decrease in pH measurement is expected as the AP does have organic acids present (Table 2) with 1242 mg/100 g of malic acid. Kęska et al. (2024) found in their research that the AP in their pork shoulder experiment affected the pH. However, over the course of the storage trial, the authors pointed out that the pH values did fluctuate. It was also demonstrated that the pH marginally decreased between treatment groups in the research conducted by Pollini et al. (2022) with beef burgers, but over the course of storage, the pH remained the same.

The crude protein measurements were influenced by the degree of inclusion of the hydrated AP, due to the reduction of the beef portion. A similar reduction was reported previously in studies aimed at investigating the inclusion of waste materials in meat products. For example, Grispoldi et al. (2022) incorporated dried AP into a salami product and observed a 5.6% decrease in protein content in the group with 14% dried AP, compared to the control. Further, Sun et al. (2022) incorporated hempseeds into chicken sausages, and despite the hemp having a 49.8% protein content, the total protein content decreased with the addition of hempseed. Further research is now warranted to investigate how a decrease in protein would affect consumer perception of hybrid meat products.

### 3.3 Objective Analysis of Cooked Meatballs

The Hunter Lab system protocol is a common measure of the color of food products. It uses L* as a lightness measurement, a* as a redness/greenness measurement, and b* as a yellowness/blueness measurement (Grasso et al. 2019). The Hunter Lab system has the Δ (delta) E calculation, which allows for an overall comparison of the color of samples. This is seen in Table 4 with a difference of 0–3 from baseline being undetectable to the human eye. A difference greater than 3 is obvious to the human eye.

**TABLE 4:** Objective analysis of the meatball with added apple pomace (AP) treatments through color, texture and yield.

Parameter measured	Control	10% AP	20% AP
Internal color (*n* = 5)			
L*	51.36 ± 0.67^a^	46.92 ± 1.34^a^	47.61 ± 0.71^a^
a*	23.51 ± 0.54^c^	13.72 ± 1.19^b^	9.08 ± 0.61^a^
b*	11.13 ± 0.27^a^	12.17 ± 0.31^a^	14.35 ± 0.2^b^
ΔE	N/A	10.81	15.26
External color (*n* = 5)			
L*	33.78 ± 0.92^a^	31.37 ± 1.46^a^	34.88 ± 1.06^a^
a*	12.24 ± 0.87^b^	10.53 ± 0.62^ab^	9.33 ± 0.38^a^
b*	11.91 ± 0.42^a^	12.00 ± 0.54^a^	13.25 ± 0.43^a^
ΔE	N/A	2.96	3.38
Texture (*n* = 5)			
Hardness (*N*)	8.52 ± 1.17^a^	8.65 ± 0.90^a^	7.46 ± 0.97^a^
Cohesiveness	0.84 ± 0.00^a^	0.79 ± 0.02^a^	0.66 ± 0.13^a^
Chewiness (mJ)	4.41 ± 0.02^a^	4.25 ± 0.03^a^	3.80 ± 0.39^a^
Yield (*n* = 10)			
Percentage lost (%)	−21.73 ± 1.58^b^	−28.88 ± 0.59^a^	−26.13 ± 0.90^a^

*Note:* The letter subscripts with different values in the same row are significantly different at *p* < 0.05.

As shown in Table 4, internal redness (a*) significantly decreased (*p* < 0.05) with increasing AP inclusion, with the following values (arbitrary units): control = 23.51; 10% AP = 13.72; and 20% AP = 9.08. All meatballs were cooked to 71°C, so differences were not due to undercooking (see Figure S2). A likely explanation is the Maillard reaction: fructose, a reducing sugar present in AP (Table 2), reacts with proteins during cooking to produce browning (Chansataporn et al. 2019).

The ΔE scores between treatments exceeded 3, indicating that color changes would be noticeable to consumers. Internal color scores for the 10% and 20% AP treatments were 10.81 and 15.26, respectively, confirming this visible difference. These findings suggest that AP contributes to a more consistent internal browning, adding potential visual appeal to meat‐based products like beef meatballs.

The external meatball color differed minimally across the treatments and the colors measured. There was only one significant difference (*p* < 0.05) between the treatments in the external color measurements. The difference in overall external color compared to the control of the treated meatballs was 2.96 for the 10% treatment group and 3.38 for the 20% treatment group, according to the Hunter Lab system. Since the ΔE values for external color measurements are close to 3, the ability of the human eye to detect differences between treatments is difficult. This is a positive observation, as Grasso and Goksen (2023) completed a review of meat hybrid products and found that consumers who eat meat are more likely to accept hybrid products with minimal changes, like external color, if the health marketing appeals to them. AP as an ingredient will not significantly affect the external color of cooked meatballs, and this is positive for consumer acceptance.

There were no significant differences (*p* > 0.05) among the four measurements of texture, hardness, cohesiveness, and chewiness, among the three treatment groups (Table 4). The water holding capacity of meat has a large influence on the texture according to Zhao et al. (2021). Table 4 shows the percentage weight loss of the meatballs post cooking. This weight loss is linked to the amount of water and solutes that are evaporated or leached from the meatball during the cooking process (Lecomte et al. 1993). The yield analysis indicated a significant difference (*p* < 0.05) in the percentage loss between the control and the treatment groups. The control had a percentage loss of −21.73%, while the 20% AP inclusion was −26.13% as seen in Table 4. However, the results from Henning et al. (2016) demonstrate that the addition of fiber into the meat matrix of their pork sausages was beneficial to yield percentages. This difference is theorized to be due to the hydration of the AP used in this study.

### 3.4 Sensory Acceptance of Meatball Treatments

**FIGURE 2:**
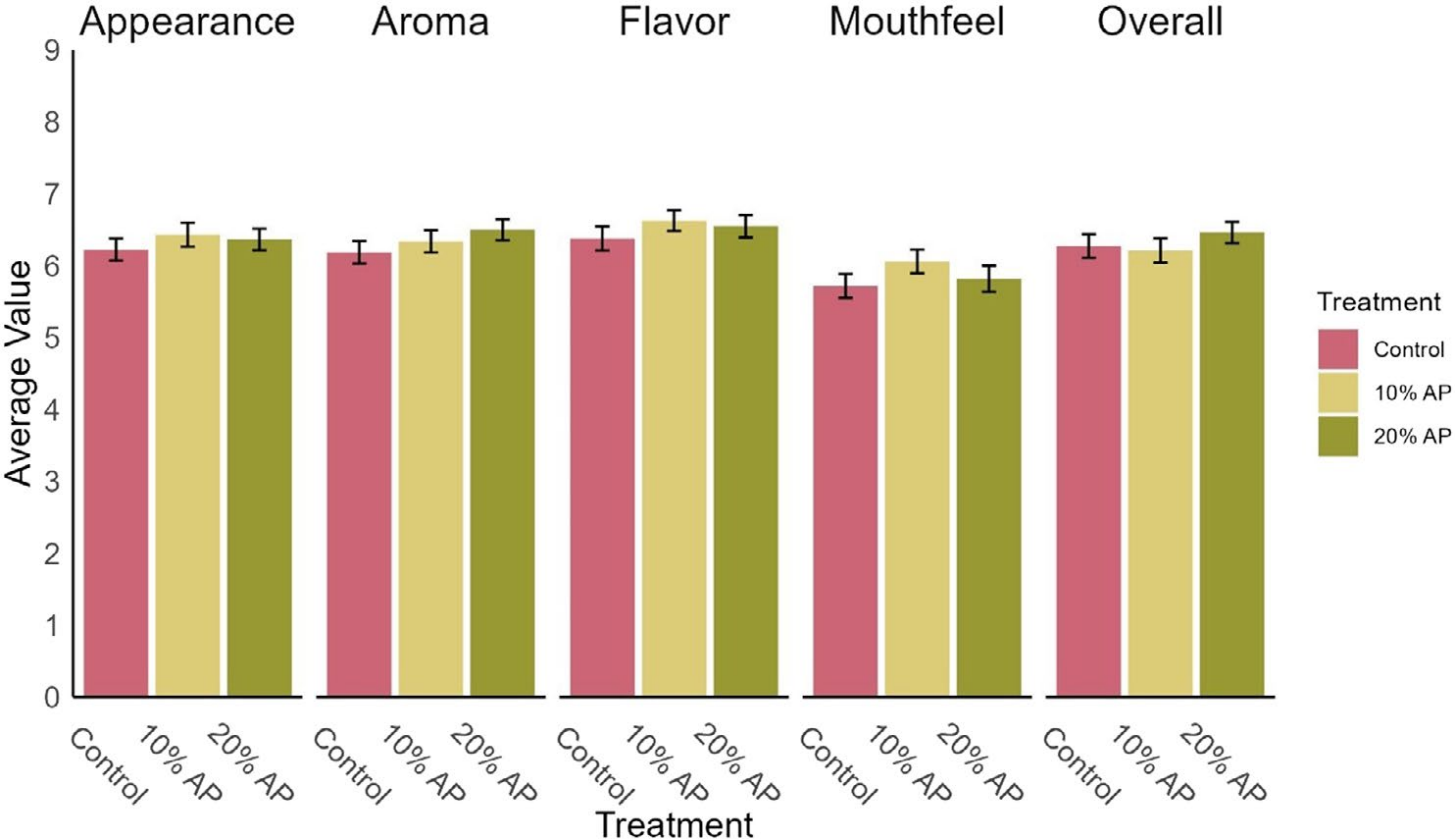
Ratings of likeness of different sensory properties of the three treatments of meatballs with apple pomace (AP) added, on a 9‐point hedonic scale.

**FIGURE 3:**
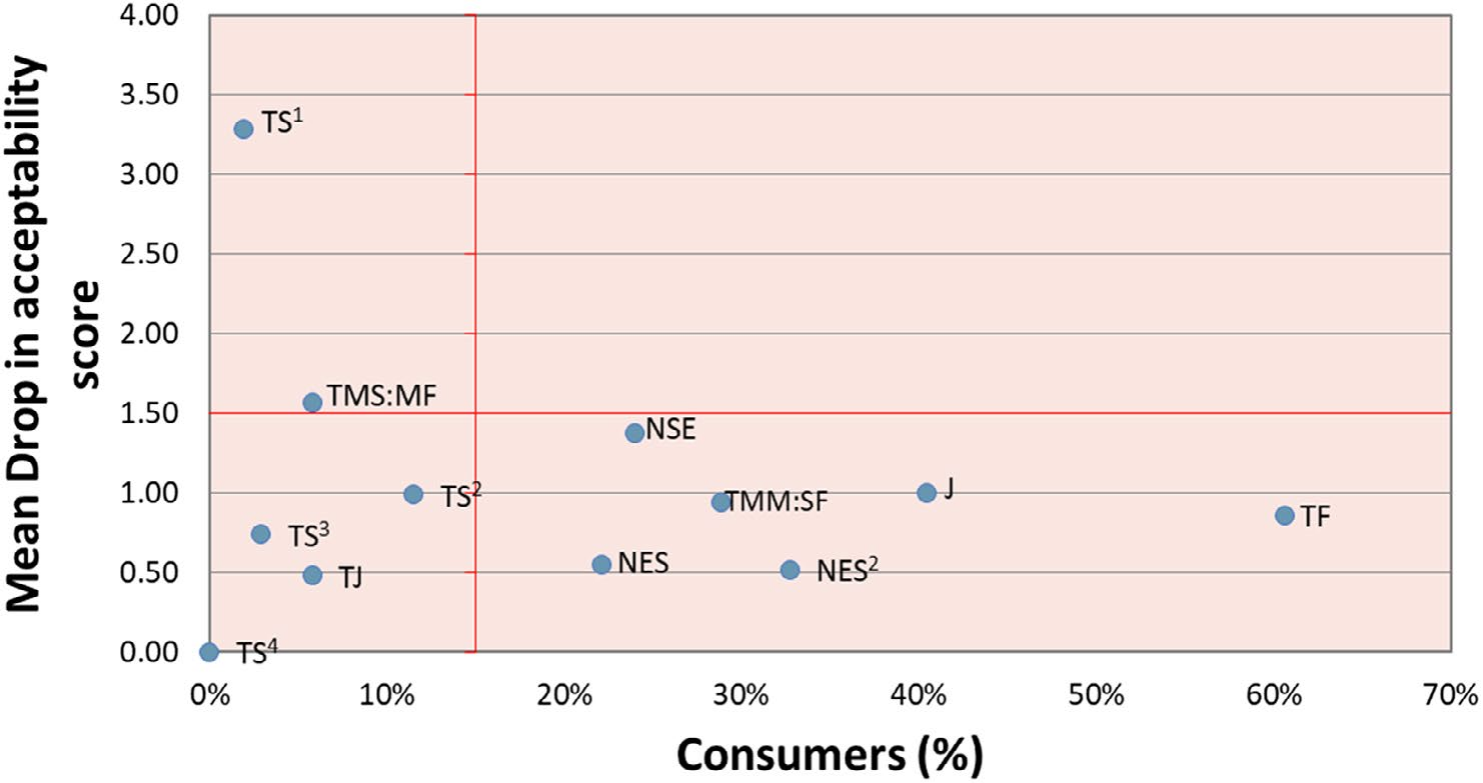
Penalty analysis report for the 10% AP meatball treatment group. The abbreviations correspond to the attribute that affected likability. J, Juiciness; NES, Not enough sourness; NES^2^, Not enough sweetness; NSE, Not salty enough; TF, Texture firm; TJ, Too juicy; TMM:SF, Too much meat; Sweet flavor; TMS:M, Too much sweet, Meat flavor; TS^1^, Texture soft; TS^2^, Too salty; TS^3^, Too sweet; TS^4^, Too sour.

The sensory analysis included 104 participants. All the participants sampled all three meatballs (Control, 10% AP, 20% AP) in the same session. Previously, AP had been tested with different meat mixes; however, no study using AP, to the authors’ knowledge, has used as many participants as possible for sensory analysis with trained or consumer assessors. The main results of the sensory analysis are presented in Figures 2, 3, and Table 5.

**TABLE 5:** Mean rank scores of meatball samples based on participant preference (1 = most preferred, 3 = least preferred).

	Control	10% AP	20% AP
Rank 1	35.36^a^	37.34^a^	31.30^a^
Rank 2	26.21^a^	35.36^a^	42.43^a^
Rank 3	42.43^a^	31.30^a^	30.26^a^
Rank Sum	213^a^	200^a^	205^a^

*Note:* Statistical analysis was conducted using Friedman's Two‐Way ANOVA by ranks (*n* = 104). No significant differences were found between treatments (*p* = 0.659; confidence = 34%).

Figure 2 shows mean sensory scores for appearance, aroma, flavor, mouthfeel, and overall liking across treatment groups. Although the 10% AP treatment received the highest average scores for appearance, flavor, and mouthfeel, and the 20% AP treatment scored highest for aroma and overall liking, these differences were not statistically significant. Friedman tests revealed no significant differences across treatments for any of the five sensory characteristics (*p* > 0.05), indicating that the inclusion of 10% or 20% apple pomace did not significantly affect consumer perception compared to the control.

There have been previous studies that have used plant ingredients and had negative sensory outcomes. An example of this would be in rice bran and pea fiber meatballs that were tested by Kehlet et al. (2017). The authors reported significant differences (*p* < 0.05) with less intensity of the meatball flavor and an increase in both the grainy texture and the meatball aroma across both treatments. This would indicate that no significant differences, as seen in Figure 2, are a positive outcome for this study, as fiber ingredient addition can cause a dislike of the product, especially at a level of 20% inclusion.

Also presented in Figure 2 is the measurement of the aroma characteristic in the sensory evaluation. The treatment groups had a better trend of aroma scores than the control. This aroma result contributed to the overall liking ranking of the 20% AP being higher than the control or the 10% AP treatment group. Similar results were found when AP was used as an ingredient in shortbread cookies, and the sensory properties, including aroma, improved with the inclusion of AP (Radzymińska et al. 2017).

The mouthfeel average hedonic scale scores were the lowest of any characteristic. There was an upward trend of 10% in the AP treatment group in terms of mouthfeel scores, with a score of 6.06 on average (Table S2). A penalty analysis identifies how deviations from an ideal sensory attribute—such as texture, flavor, or appearance—impact overall liking scores. It allows pinpointing which sensory characteristics, when rated as “too much” or “too little” by consumers, significantly lower the product's acceptability. Figure 3 shows that both soft and firm textures had the greatest negative impact on overall liking scores compared to other attributes, indicating texture played a key role in consumer perception of the meatballs. It has been stated in previous research that dietary fiber added to meat can improve texture and the rheological properties of the meat (Kim and Paik 2012; Talukder 2015). The mouthfeel ratings for the 10% AP meatballs were comparable to those of the control and the 20% AP treatments, suggesting that this level of inclusion did not negatively impact consumer perception of texture.

At the conclusion of the sensory test, participants ranked the three meatball samples in order of preference. Although the control treatment appeared most frequently in the third‐place rankings, Friedman's Two‐Way ANOVA by ranks indicated no statistically significant differences in overall preference between treatments (*p* = 0.659). The 10% AP inclusion was ranked first the most often, and the 20% AP inclusion was then most often ranked as the second most preferred sample by the participants. This ranking of the treatments (10% AP, 20% AP) is higher than the control, demonstrating that there is a format in which meat reduced products can be what the consumer prefers. This can only be achieved if the food product meets taste and flavor expectations; if unmet, these can be major obstacles to the growth of the meat reduction market (Caputo et al. 2023).

A penalty analysis is used to assess which attributes of a food product affect likeability. The red lines in Figure 3 represent thresholds commonly used in penalty analysis interpretation. The vertical line at 20% indicates the minimum percentage of consumers who must report an issue for it to be considered practically relevant, while the horizontal line at a 1.5‐point drop in acceptability reflects a meaningful decrease in liking. Attributes that fall into the top‐right quadrant—exceeding both thresholds—are considered key drivers of consumer dissatisfaction and should be prioritized for reformulation. The sensory attributes used are listed below Figure 3 and were assessed with hedonic and JAR scales, from sensory evaluation.

These scales can then be used to determine which attributes affected likeability with what percentage of consumers (Osunbade et al. 2025). The 10% AP treatment had the most number 1 rankings of any of the treatments (Table 5). The penalty analysis for the 10% AP meatballs is shown in Figure 3. The X axis of the penalty analysis shows the percentage of consumers that the attribute plotted was affected by. The Y axis is the mean drop that the attribute caused in the dislike of the product. An example of an attribute that caused a likeness drop in a small number of participants was the texture being “too soft.” A sensory attribute that did not cause a severe drop in liking but affected over 30% of participants, was the “not enough sweetness” attribute. The two attributes used as examples would not need to be considered by a product development team to be changed as they are not within the quadrant that means a high percentage of consumers found a characteristic with a high level of unacceptability (Ares et al. 2014).

The “too much sweet to meat flavor ratio” caused a mean drop of over 1.5 in liking but only affected less than 10% of consumers. The juiciness attribute of the meatball caused a mean drop of over one in likeness but affected 40% of consumers. The attributes that did not contribute to participants disliking the sample were “too sour,” “too juicy,” “too sweet,” or “too salty.” As noted by Miller (2020) high‐ intensity flavors are not necessarily perceived negatively in meat products, which may help explain these findings.

Results determined by Neville et al. (2017) demonstrated that for their pork sausages the texture was one of the most important attributes for consumer acceptability. The importance of texture in meat products has been demonstrated in the results seen in Figure 3 as the texture firm affected the most consumers and texture soft affecting acceptability the most in the 10% AP meatball.

In summary, the scope of the current study was to analyze AP as a fiber that is beneficial to consumers and could be included in food products like meat. The polyphenols and the fiber presented in Table 2 show the value that can be added from AP as a food ingredient. The consistently higher ranking of the inclusion treatments demonstrates AP as a viable ingredient for consumer acceptance in meat products like meatballs.

## 4 Conclusion

The current study presented AP analysis, as well as the objective and subjective analysis of beef meatballs fortified with 10% and 20% hydrated AP. The AP chemical analysis determined that there were polyphenols and fiber present in the AP. This adds to the growing body of research demonstrating the health potential of AP as a bioactive, food product ingredient. AP influenced the proximate composition of the meatballs by reducing protein content and increasing dietary fiber. It also affected internal color, resulting in a browner appearance, which may be perceived as either a benefit or a drawback depending on consumer preference. The hedonic scale and rank summary results showed no significant differences between the meatball samples. As the inclusion of AP did not affect the meatball characteristic scores or rankings, it suggests that AP could be a potentially viable ingredient for inclusion in meat‐based food products, warranting further exploration. Further research should find at what point in AP inclusion it becomes unacceptable to consumers as well as investigating other food products in which AP could be included as a bioactive, sustainable ingredient, and to minimize its environmental impact. This could provide consumers with the much‐needed additional dietary fiber that is lacking in the western diet.

## Author Contributions

**Peter Gracey:** data curation (equal), formal analysis (equal), investigation (equal). **Olga I. Padilla‐Zakour:** conceptualization (equal), methodology (equal), writing – review and editing (equal). **Elad Tako:** conceptualization (equal), methodology (equal), project administration (equal), resources (equal), supervision (equal), writing – review and editing (equal).

## Supporting information


**Table S1:** fsn370955‐sup‐0001‐TableS1‐S2‐FigureS1‐S7.docx. *P*‐values from Friedman tests for sensory attributes across treatment groups
**Figure S1:** Settings used for freeze‐drying apple pomace
**Figure S2:** The internal color of the three treatment groups of meatballs. From left to right: Control, 10% Apple Pomace, and 20% Apple pomace.
**Figure S3:** Percentage of study participants purchase intents for each treatment group
**Figure S4:** The percentage the participants chose a food claim as important
**Figure S5:** Penalty analysis of the control group.
**Figure S6:** Penalty analysis of 20% AP treatment group.

## Data Availability

The datasets generated and analyzed during this study are available from the corresponding author upon reasonable request.
