# Supplementary material for: Sensory Acceptance and Physicochemical Properties of Beef Meatballs Fortified With Apple ( Malus domestica ) Pomace
Source: Food Sci Nutr. 2025 Sep 12;13(9):e70955. doi: 10.1002/fsn3.70955 (PMC12431853; doi:10.1002/fsn3.70955)
Supplement: Supplementary file 1 — Table S1: fsn370955‐sup‐0001‐TableS1‐S2‐FigureS1‐S7.docx. P‐values from Friedman tests for sensory attributes across treatment groups Figure S1: Settings used for freeze‐drying apple pomace Figure S2: The internal color of the three treatment groups of meatballs. From left to right: Control, 10% Apple Pomace, and 20% Apple pomace. Figure S3: Percentage of study participants purchase intents for each treatment group Figure S4: The percentage the participants chose a food claim as important Figure S5: Penalty analysis of the control group. Figure S6: Penalty analysis of 20% AP treatment group. [file FSN3-13-e70955-s001.docx]

Supplementary materials

**Table S1**: P-values from Friedman tests for sensory attributes across treatment groups

| Characteristic and Groups Compared | p- value |
| --- | --- |
| Appearance | 0.33 |
| Aroma | 0.26 |
| Flavor | 0.45 |
| Mouthfeel | 0.47 |
| Overall | 0.44 |

**Table S2:** Average hedonic scale scores

| Treatment Group | Characteristic | Average value | Standard error |
| --- | --- | --- | --- |
| Control | Appearance | 6.22 | 0.15 |
| Control | Aroma | 6.18 | 0.16 |
| Control | Flavor | 6.38 | 0.17 |
| Control | Mouthfeel | 5.72 | 0.16 |
| Control | Overall | 6.27 | 0.16 |
| 10AP | Appearance | 6.43 | 0.17 |
| 10AP | Aroma | 6.34 | 0.15 |
| 10AP | Flavor | 6.63 | 0.14 |
| 10AP | Mouthfeel | 6.06 | 0.17 |
| 10AP | Overall | 6.21 | 0.17 |
| 20AP | Appearance | 6.37 | 0.15 |
| 20AP | Aroma | 6.50 | 0.14 |
| 20AP | Flavor | 6.55 | 0.16 |
| 20AP | Mouthfeel | 5.82 | 0.18 |
| 20AP | Overall | 6.46 | 0.15 |


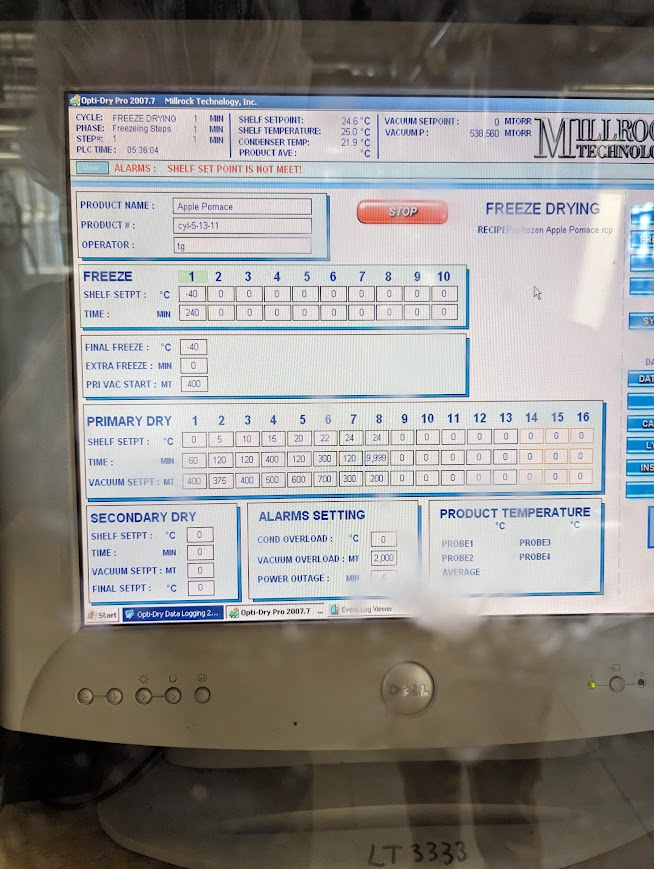


**Figure S1:** Settings used for freeze-drying apple pomace


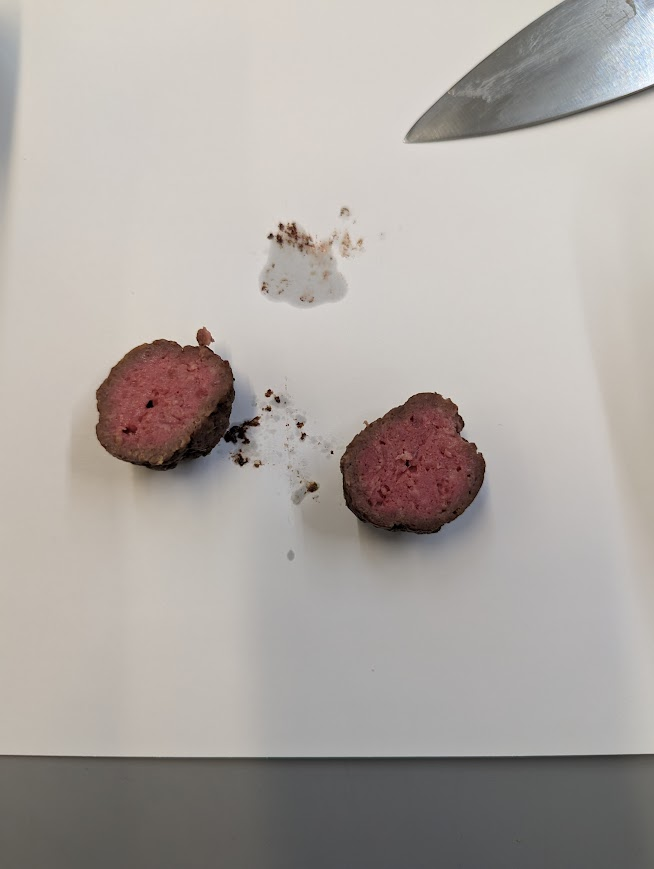

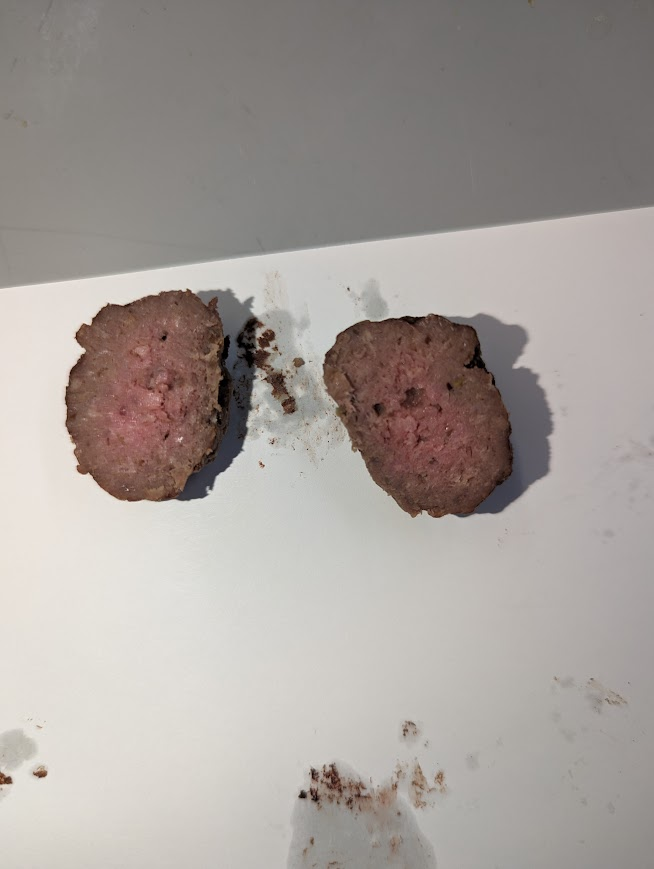

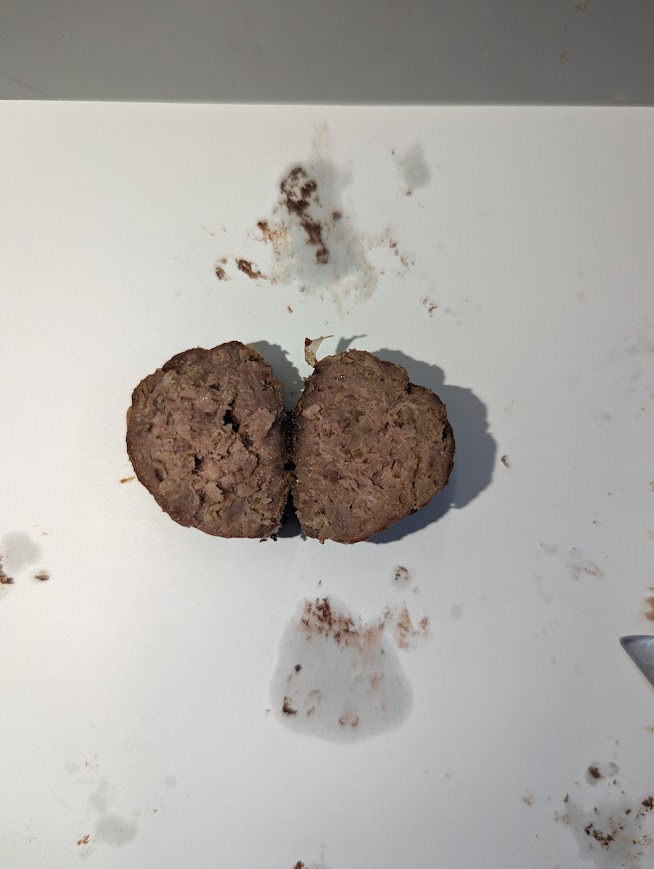


**Figure S2:** The internal color of the three treatment groups of meatballs. From left to right: Control, 10% Apple Pomace, 20% Apple pomace.


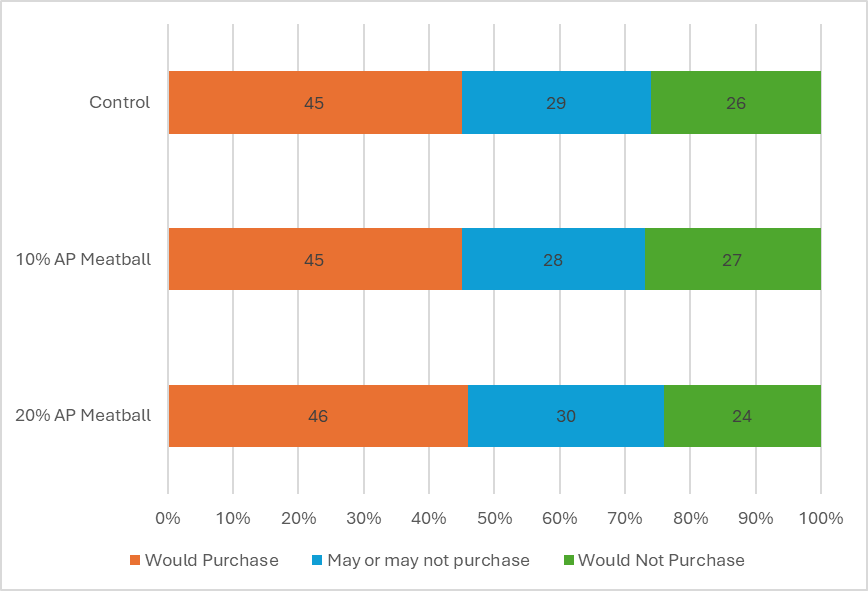


**Figure S3:** Percentage of study participants purchase intents for each treatment group.


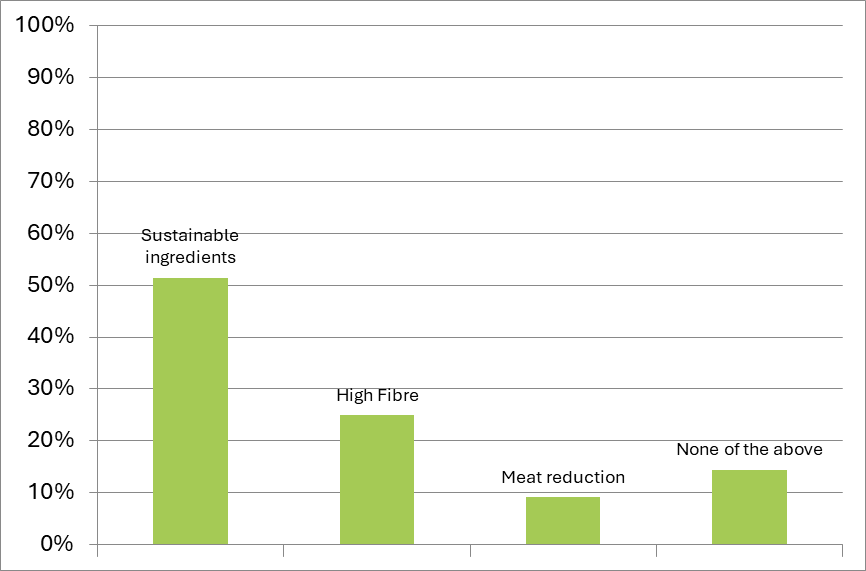


**Figure S4:** The percentage the participants chose a food claim as important.


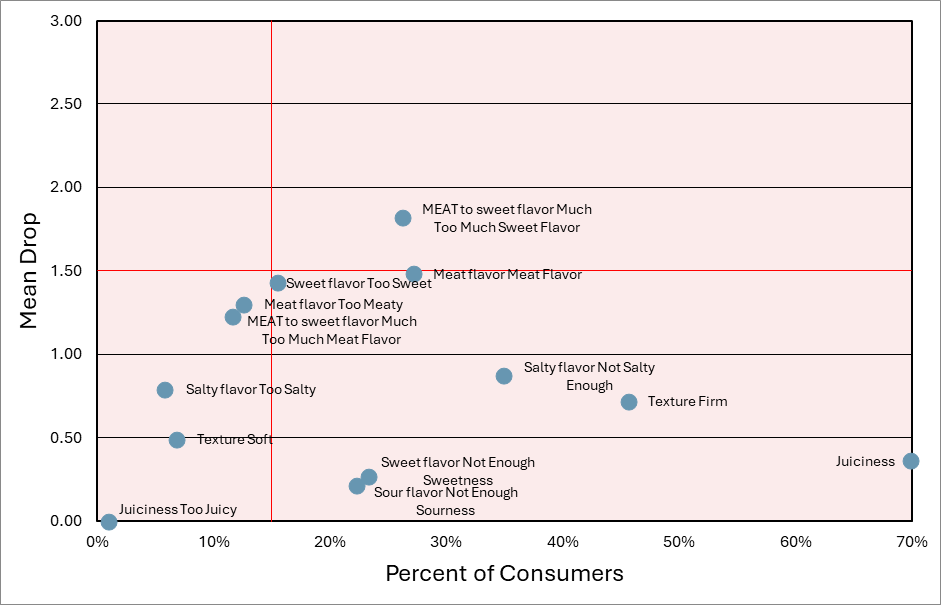


**Figure S5:** Penalty analysis of the control group.


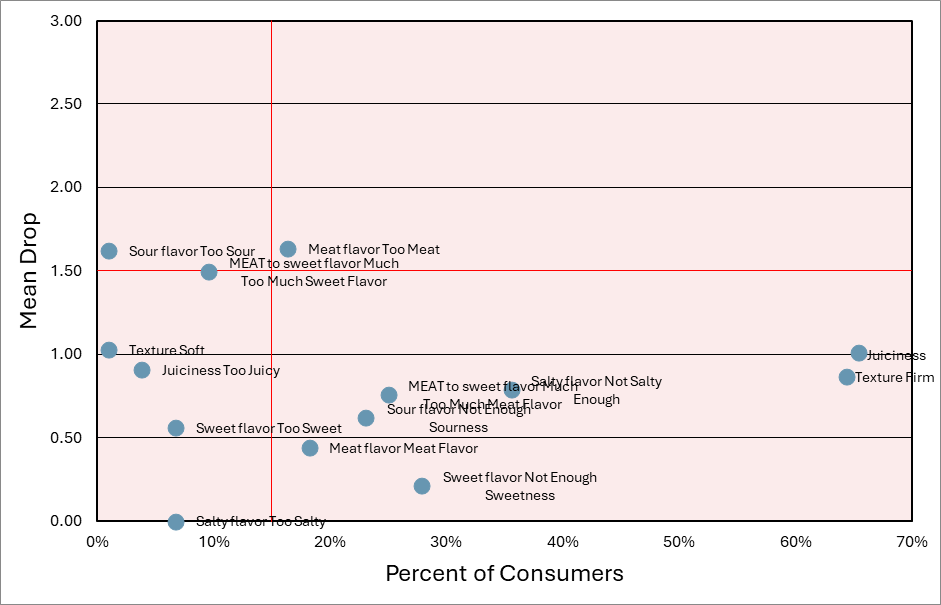


**Figure S6:** Penalty analysis of 20% AP treatment group

Sensory questions

Before tasting the sample please answer the following question

How would you rate this test samples Appearance? (please select one response)

- Like it extremely
- Like it very much
- Like it moderately
- Like it slightly
- Neither like nor dislike it
- Dislike it slightly
- Dislike it moderately
- Dislike it very much
- Dislike it extremely

Please now take a bite of the sample

Thinking about the sample overall would you say you… ? (please select a response)

- Like it extremely
- Like it very much
- Like it moderately
- Like it slightly
- Neither like nor dislike it
- Dislike it slightly
- Dislike it moderately
- Dislike it very much
- Dislike it extremely

If this product were available to you in a store where you usually shop at a price that you typically pay, and from the brand that you typically buy, would you say you would…? (Select one response)

- Definitely would purchase
- Probably would purchase
- May or may not purchase
- Probably would not purchase
- Definitely would not purchase

How well or not does this test sample meet your expectations for a meatball? (Select one response)

- Definitely meets my expectations
- Probably meets my expectations
- Neither does nor does not meet my expectations
- Mostly does not meet my expectations
- Definitely does not meet my expectations

How would you rate the aroma of this sample? (Please select one response)

- Like it extremely
- Like it very much
- Like it moderately
- Like it slightly
- Neither like nor dislike it
- Dislike it slightly
- Dislike it moderately
- Dislike it very much
- Dislike it extremely

How would you rate the flavor of this sample?

- Like it extremely
- Like it very much
- Like it moderately
- Like it slightly
- Neither like nor dislike it
- Dislike it slightly
- Dislike it moderately
- Dislike it very much
- Dislike it extremely

Thinking about the meat flavor, would you say it was… ? (Please select one)

- Not enough meat flavor
- Somewhat meat flavored
- Just about right amount of meat flavor
- Somewhat too meat flavored
- Much too meat flavored

Thinking about the sweet flavor, would you say it was …? (please select one response)

- Not enough sweetness
- Somewhat sweet
- Just the right amount of sweetness
- Somewhat too sweet
- Much too sweet

Thinking about the sour flavor, would you say it was… ? (Please select one response)

- Not enough sourness
- Somewhat sour
- Just about right level of sourness
- Somewhat too sour
- Much too sour

Thinking about the level of saltiness, would you say it was…? (Please select one response)

- Not salty enough
- Somewhat not salty enough
- Just about right level of saltiness
- Somewhat too salty
- Much too salty

Thinking about the meat to sweet taste ratio, would you say it was…? (please select one response)

- Much too much meat flavor
- Somewhat too much meat flavor
- Just about right ratio of meat to sweet flavor
- Somewhat too much sweet flavor
- Much too much sweet flavor

Do you find an aftertaste with this sample?

- Yes
- No

Thinking about the mouthfeel of this sample, would you say you…?

- Like it extremely
- Like it very much
- Like it moderately
- Like it slightly
- Neither like nor dislike it
- Dislike it slightly
- Dislike it moderately
- Dislike it very much
- Dislike it extremely

Thinking about the juiciness of this sample, would you say it was…? (please select one response)

- Not at all juicy/ very dry
- Somewhat juicy
- Just the right amount of juiciness
- Somewhat too juicy
- Much too juicy

Thinking about the texture of the sample, would you say it was…?

- Too soft
- Soft
- Just the right amount of firmness
- Firm
- Too firm

If you have any additional thoughts or suggestions about this test product, please let us know below. If not, please type in “none”.

_________________________________________________

You have just consumed two beef meatball samples with a meat reduction of 10 and 20%. This reduction was made from waste upcycled from the cider industry.

Please consume the rest of the samples. Please rank the samples in the order you prefer them from most to least. Start by selecting the product code you liked the most, followed by clicking the product code you liked second, continuing until the final product code you click in the one you liked the least. If you would like to change the order of your responses, click the reset button in the lower right- hand corner.

447 181 883

Can you please descried why you ranked the samples in the order?

________________________________________________

Which of these claims on food products are most important to you?

- Sustainable ingredients
- High Fiber
- Meat reduction
- None of the above

Are you aware of polyphenols and/or Bioactives and their health benefits?

- Yes
- No

With knowledge you have would a “polyphenol” and/or “bio actives presents” claim be a selling feature to you?

- Yes
- No

Thinking about beef meatballs as a product category or food type, would you say you are…? (Please select one response)

- Not at all familiar
- Not too familiar
- Somewhat familiar
- Moderately familiar
- Very familiar

Thinking about apple derived products as a food/ beverage type, would you say you are…? (Please select one response)

- Not at all familiar
- Not too familiar
- Somewhat familiar
- Moderately familiar
- Very familiar

What’s your age?

________________

What is your gender? (Select one)

- Male
- Female
- Non-conforming
- Prefer not to say

**Figure S7:** Sensory questions asked of the participants
